# Raman characterization of Cu_2_ZnSnS_4_ nanocrystals: phonon confinement effect and formation of Cu_*x*_S phases†

**DOI:** 10.1039/c8ra05390a

**Published:** 2018-08-31

**Authors:** Ye. Havryliuk, M. Ya. Valakh, V. Dzhagan, O. Greshchuk, V. Yukhymchuk, A. Raevskaya, O. Stroyuk, O. Selyshchev, N. Gaponik, D. R. T. Zahn

**Affiliations:** V. E. Lashkaryov Institute of Semiconductor Physics, Nat. Acad. of Sci. of Ukraine 03028 Kyiv Ukraine; L. V. Pysarzhevsky Institute of Physical Chemistry, Nat. Acad. of Sci. of Ukraine 03028 Kyiv Ukraine alstroyuk@ukr.net; Physical Chemistry, TU Dresden 01062 Dresden Germany; Semiconductor Physics, Chemnitz University of Technology 09107 Chemnitz Germany

## Abstract

A Raman spectroscopic study of Cu_2_ZnSnS_4_ (CZTS) nanocrystals (NCs) produced by a “green” synthesis in aqueous solutions is reported. Size-selected CZTS NCs reveal phonon confinement that manifests itself in an upward shift of the main phonon peak by about 3–4 cm^−1^ by varying the NC diameter from 3 to 2 nm. A non-monotonous shift and narrowing of the main peak are attributed to the special shape of the phonon dispersion in this material. Moreover, the method of sample preparation, the nature of the supporting substrate and the photoexcitation regime are found to crucially influence the Raman spectra of the CZTS samples. Particularly, the possible oxidation and hydrolysis of CZTS NCs with the concomitant formation of a Cu–S phase are systematically investigated. The nature of the film support is found to strongly affect the amount of admixture copper sulfide phases with the Cu_2−*x*_S/CuS content being the highest for oxidized silicon and glass and notably lower for ITO and even less for gold supports. The effect is assumed to originate from the different hydrophilicity of the supporting surfaces, resulting in a different morphology and surface area of the NC film exposed to the atmosphere, as well as the degree of the NC oxidation/hydrolysis. The amount of copper sulfide increases with the laser power. This effect is interpreted as a result of photochemical/photocatalytic transformations of the CZTS NCs.

## Abstract 2

A Raman spectroscopic study of Cu_2_ZnSnS_4_ (CZTS) nanocrystals (NCs) produced by a “green” synthesis in aqueous solutions is reported.

## Graphical abstract

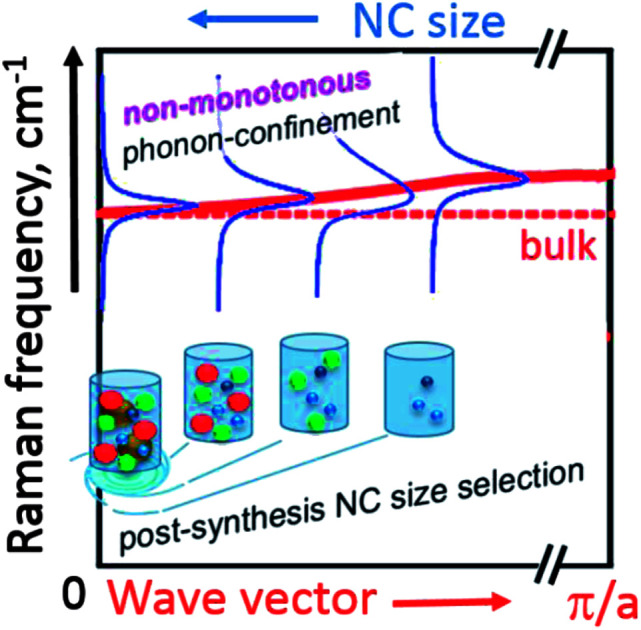

## 1 Introduction

Silicon-based solar cells currently cover approximately 85% of the total photovoltaic market with a top light conversion efficiency of 27%.^1^ Despite the inherently low light absorption efficiency in this indirect-bandgap semiconductor, it dominates the market due to the material availability and the high-quality thin film technology being available for mass production. However, exponential growth of silicon photovoltaics in the last few years has required increasingly larger areas for solar cells and huge volumes of high-quality Si for their fabrication.

The direct-bandgap chalcogenide-based compounds offer a promising solar cell alternative which benefits from high light absorption in comparison to Si.^2,3^ One such material is kesterite Cu_2_ZnSnS_4_ (CZTS). This material has a direct bandgap of 1.4–1.6 eV and a high absorption coefficient of above 10^4^ cm^−1^, and all its constituents are Earth-abundant and affordable, and have low toxicity. A power conversion efficiency of ≈13% has already been reached for small-area devices based on CZTS.^1,2^

For a further boost of the efficiency of this complex compound, a number of challenges have to be met related to the purity of the crystal structure with respect to non-stoichiometry, point defects, numerous polymorphs, and secondary phases.^4–9^

Advances in the synthesis of high quality semiconductor nanocrystals (NCs) opened new possibilities for thin-film devices,^10,11^ including third-generation solar cells,^12^ due to their tunable bandgap, efficient optical absorption, stability, and capability of multiple exciton generation.^3,11^ NCs produced by colloidal chemistry are especially promising, because they combine the tunability of optical properties by tailoring of the NC size and versatile surface functionalization.^13,14^ At the same time, colloidal NCs in the form of concentrated inks are compatible with roll-to-roll processing and modern thin film printing technologies,^11,15^ and can be obtained directly in aqueous media.^16–18^ Being more environmentally friendly than most II–VI counterparts, CZTS NCs were also intensively investigated for many applications beyond photovoltaics.^19,20^

Raman spectroscopy proved to be a very efficient diagnostic tool of the structure and composition of CZTS and related compounds.^7,9,21–29^ In many cases, it allows the CZTS polymorphs and secondary phases to be distinguished more reliably than by other conventional structural tools such as XRD, SAED, or TEM. The reasons for that are the better separation of phonon frequencies of the crystal structures in question as compared to their diffraction patterns, as well as the possibility to probe upon demand macro-, micro-, or even nanovolumes of the material.

The advantage of Raman spectroscopy becomes even more evident in the case of NCs, where additional significant broadening of XRD reflexes is contributed by NC size and size distribution.^30^ Despite this, Raman studies of CZTS NCs prepared by colloidal chemistry are relatively rare up to now,^8,13,14^ as compared to the intensively studied bulk CZTS crystals, thin CZTS films^7,9,21–29^ as well as binary and ternary chalcogenide NCs.^31–33^ Moreover, the reported results for CZTS NCs are contradictory to a certain extent. Thus, a decrease of the kesterite NC size was found to shift the frequency of the main CZTS phonon peak upwards in ref. 14, while a constant peak position was observed for NCs strongly differing in the average size,^13,34^ and even an opposite (downward) shift was predicted by calculations.^24^ A very non-monotonous variation of the Raman peak positions with NC size was also observed.^35^

Typically, the main Raman peak of kesterite (KS) CZTS NCs is reported at 336–339 cm^−1^, ^13,30,35–39^ matching well the values reported for bulk CZTS crystals and films.^6,28,40–42^ Nevertheless, frequencies in the range of 330–334 cm^−1^ can be also found in the literature for KS CZTS NCs,^34,43–46^*i.e.* in the same range where the strongest Raman features of wurtzite (WZ)^35,36,47,49^ and stannite CZTS NCs^40,42^ were often reported. For example, a Raman spectrum very close to the kesterite one with peaks at 334–337 cm^−1^ was reported for WZ CZTS NCs in a surface-enhanced Raman scattering study,^8^ while the ordinary Raman spectra in the same work revealed the main peak at 326 cm^−1^. The main Raman frequency was also reported to be 10 cm^−1^ below that of bulk KS-CZTS.^15^ These examples indicate that not only a NC size effect but also other factors affecting the phonon frequencies should be taken into account in such a complex material as CZTS.

In particular, the possible formation of secondary phases of binary and ternary chalcogenides and intrinsic defect structures is typically considered for CZTS NCs.^5,8^ One of the most probable secondary phases in CZTS is Cu_2−*x*_S.^23^ The identification of this phase is complicated by the availability of several stable Cu_2−*x*_S modifications corresponding to different *x*, which have dramatically different structural, physical, and chemical properties.^32,48^ The presence of secondary phases in CZTS depends quantitatively and qualitatively on the technological conditions and post synthesis treatments.^4,5^ A variation of either chemical conditions (*e.g.* concentration of reagents and ligands) or physical conditions (*e.g.* temperature) is typically used to tune the size of CZTS NCs. However, changing such crucial factors of the NC formation can influence not only the NC size but also the NC shape and the lattice type as well as the content of secondary phases – the factors that are not always (or rarely) considered in the studies of the size-dependent CZTS NC properties.

The aim of the present work is the investigation of the Raman spectroscopic properties of CZTS NCs prepared by a colloidal synthesis directly in water as well as size-selected CZTS NCs produced by the fractionation of such colloids. Such approach allows the size effects in the phonon spectra of CZTS NCs to be separated from those related with secondary phases and intrinsic defect structures because NC samples with different mean size were obtained from the single ensemble and therefore formed in identical conditions similarly to those reported by us earlier for the ternary Ag–In–S NC system.^18,49^

Here, we separated up to nine fractions of NCs and characterized them by multi-wavelength Raman spectroscopy. Additionally to size-dependent data, we report striking differences in the Raman spectra of CZTS NCs deposited on different substrates. This fact is usually overlooked in the literature and makes the comparison of different reports quite questionable. We established that the exposure of CZTS NCs to ambient moisture and air results in a partial hydrolysis/oxidation of CZTS with the formation of Cu_2−*x*_S as a secondary phase and transformation of the remaining CZTS phase into a disordered kesterite one. According to our findings, the latter transition is additionally stimulated by the laser illumination during Raman measurements, similar to the effect observed by us earlier for bulk CZTS crystals.^6,7^

Consequently, we established the combination of the drying rate and environment as well as measurement conditions which do not lead to a deterioration of the CZTS NCs. Besides the importance of unveiling the peculiarities of secondary phase formation in CZTS NCs on its own, the results obtained in this work are important for the correct interpretation of the inherent CZTS phonon features, required for reliable structural diagnostics of this promising NC material and understanding its fundamental properties.

## 2 Experimental

### 2.1 Preparation of CZTS NCs

Colloidal CZTS NCs were produced from a mixture of mercaptoacetate (MA) complexes of Cu(ii), Zn(ii), and Sn(iv) reacting with sodium sulfide in water at 95–98 °C. The Sn(iv)-MA complex was formed *in situ* by oxidizing a Sn(ii)–MA complex in air oxygen. In a typical synthesis, 0.3 mL 0.1 M Cu(NO_3_)_2_ solution, 0.34 mL 0.5 M SnCl_2_ (with 4.0 M NaOH) solution, and 0.15 mL 1.0 M Zn(NO_3_)_2_ solution were added to 6.0 mL deionized (DI) water under vigorous stirring followed by 3.0 mL aqueous 1.0 M mercaptoacetic acid (MAA) and 0.3 mL 1.0 M NaOH solution. The concentration of metals in the formed solution was [Cu^II^] = 0.030 M, [Sn^IV^] = [Zn^II^] = 0.015 M. Finally, 0.3 mL 1.0 M Na_2_S solution was added and the resulting mixture was maintained at 95–98 °C for 10 min. The as-formed CZTS NCs were precipitated by adding an equal volume of 2-propanol, separated from the supernatant solution by centrifugation and redispersed in DI water.

### 2.2 Size selection of CZTS NCs

The size-selected CZTS NCs were produced from the colloidal ensemble by selective precipitation with a non-solvent – 2-propanol.^18,49^ Typically, 0.5 mL of 2-propanol was added to 10.0 mL colloidal CZTS solution resulting in a partial NC precipitation. The mixture was centrifuged at 3000 rpm for 5 min, the NC precipitate was separated from the supernatant solution and re-dispersed in 0.5 mL DI water yielding fraction F1. This fraction was typically discarded because it contains the largest NCs and unreacted species. The supernatant solution was again mixed with 0.4 mL 2-propanol and the precipitation/redispersion procedure was repeated for 4 times with the same 2-propanol volume resulting in fractions F2–F5. Fractions F6 and F7 were produced by adding 1.0 mL 2-propanol, fractions F8 and F9 with 2.0 mL and 3.0 mL 2-propanol, respectively. Representative fractions F2, F6, and F8 were selected for further characterizations and optical studies.

The CZTS NCs were deposited on a set of substrates (glass, indium tin oxide (ITO) glass, silicon wafers with oxidized surface and gold) for optical studies and dried in different conditions. A detailed description of various modes of sample preparations is provided in ESI.† The Raman spectra were taken from three different spots of each sample. Deposition on different substrates was performed with several inks (NC solutions), synthesized with minor differences in the protocol or reagents used. A set of representative samples showing typical spectral features were selected for the detailed discussion in the present paper.

### 2.3 Instruments

Absorption spectra were recorded using a UV-vis spectrophotometer Cary 60 in standard 1.0 cm optical quartz cuvettes using DI water as a reference. Raman spectra were excited by the 514.7 nm line of diode pumped solid state laser (cobolt) or 632.8 nm He–Ne laser line and registered with a spectral resolution of about 2 cm^−1^ using a LabRam HR800 micro-Raman system equipped with a liquid-nitrogen-cooled CCD detector. The incident laser power under the microscope objective (50×) was set to 0.1 mW or 0.001 mW.

X-ray diffractograms were recorded on a Bruker D2 Phaser diffractometer with a rate of 0.05° min^−1^ with monochromatized copper K_α_ irradiation. The samples were mixed with acetone (v/v = 1/1) and drop-casted on a silicon wafer (which was used as an internal standard for the evaluation of diffraction peak widths) followed by drying in a stream of nitrogen at room temperature. Transmission electron microscopy (TEM) was performed using a FEI Tecnai G2 microscope at an accelerating voltage of 300 kV. Scanning electron microscopic (SEM) and energy-dispersive X-ray spectroscopic (EDX) data were acquired using a Nova NanoSEM scanning electron microscope equipped with an EDX Bruker AXS Microanalysis setup.

## 3 Results and discussion

### 3.1 Characterization of MA-capped aqueous CZTS NCs

The mixture of MA complexes of Cu^II^, Sn^IV^, and Zn^II^ reacts with sodium sulfide at 95–98 °C resulting in very stable dark-brown colloidal MA-stabilized CZTS NCs. The NCs can be easily separated from the maternal solution by adding an equal volume of 2-propanol. The resulting precipitate can be redispersed in DI water yielding stable for months purified colloidal solutions. The syntheses starting from both Sn^II^ and Sn^IV^ precursors were found to produce identical results but the utilization of Sn^II^ is preferable as SnCl_2_ is readily dissolvable in water without noticeable hydrolysis.

**Fig. 1:**
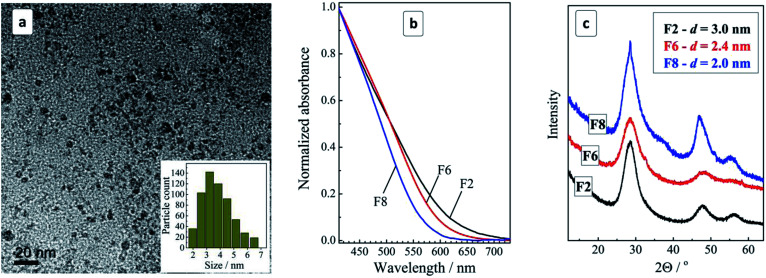
(a) TEM and size distribution (inset) of CZTS NCs in the original colloidal ensemble; (b and c) normalized absorption spectra (b) and XRD patterns (c) for the size-selected CZTS NCs in samples F2, F6, and F8. Size determination error in (c) is ±0.2 nm.

TEM showed that purified colloids contain CZTS particles with a size varying from 2 to 7 nm and the size distribution maximum at 3–4 nm (Fig. 1a). The CZTS NCs are crystalline showing a typical tetragonal kesterite structural motif (Fig. 1c) distinctly differing from Cu–S and Sn–S NC phases prepared by a similar approach from corresponding MA complexes (ESI, Fig. S1†). As will be discussed below, the CZTS NC samples also demonstrated distinct Raman spectral features characteristic for crystalline kesterite.

An EDX study of the CZTS NCs deposited on ITO showed all four elements to be evenly distributed across the sample indicating the absence of “islands” of some additional phases, in accordance with the XRD results (ESI, Fig. S2†). The sample was found to contain 13 at% Cu, 10 at% Zn, and 7 at% Sn, which is close to the expected kesterite Cu_2_ZnSnS_4_ stoichiometry. However, the Sn signal overlaps with that of In, thus possibly compromising the accuracy of the determination.

We performed a comprehensive study of the influence of synthesis parameters on the spectral properties and composition of CZTS NCs. An extended analysis of the XPS results goes beyond the scope of the present paper and will be published elsewhere. Shortly summarizing, XPS revealed copper, zinc, and tin to be present in the expected oxidation states of +1, +2, and +4, respectively. The composition of the NCs was found to deviate from the Cu_2_ZnSnS_4_ stoichiometry towards larger Cu-to-Sn atomic ratios. The exact ratios depend strongly on the history of the sample preparation.

The colloidal ensemble of CZTS NCs can be separated into a series of size-selected fractions by a measured addition of a non-solvent. The largest NCs in the ensemble precipitate first, thus allowing fractions of NCs with a decreasing size to be isolated in a step-by-step manner. The precipitates can then be redispersed in water producing stable colloidal CZTS solutions. Fig. 1b shows absorption spectra of three selected fractions of the size-selected CZTS NCs. As the fraction number increases from 2 to 8, the absorption band edge shifts to higher energies indicative of a decrease of the average NC size in the corresponding fractions.

The absorption spectra can be linearized in the coordinates of the Tauc equation for direct interband electron transitions (ESI, Fig. S2†) allowing the bandgap of the CZTS NCs in different fractions to be approximately estimated. It was found that CZTS NCs in fraction F2 are characterized by a bandgap *E*_g_ of 1.72 eV increasing to 1.85 eV for fraction F6 and to 1.98 eV for fraction F8. In all three cases *E*_g_ is much larger than the values reported for bulk Cu_2_ZnSnS_4_ kesterite (≈1.5 eV) indicating a strong spatial confinement of the charge carriers in the CZTS NCs. The size variation of the bandgap is expected for CZTS NCs smaller than ≈5 nm (ref. 13) and, therefore, the observed *E*_g_ variation indicates on the presence of smaller CZTS NCs in the studied ensemble. This conclusion is in accordance with the above discussed TEM data showing the domination of 3–4 nm CZTS in the samples under discussion.

An XRD study of the representative fractions F2, F6, and F8 showed them to share a general kesterite structural motif without additional phases (Fig. 1c). The average NC size in the fractions estimated from the broadening of the diffraction peaks using the Scherrer equation was found to decrease from 3 nm for fraction F2 to 2.4 nm for F6 and to 2.0 nm for F8.

### 3.2 Phonon Raman spectra of CZTS NCs

#### 3.2.1 Phonon spectra of unfractionated samples

**Fig. 2:**
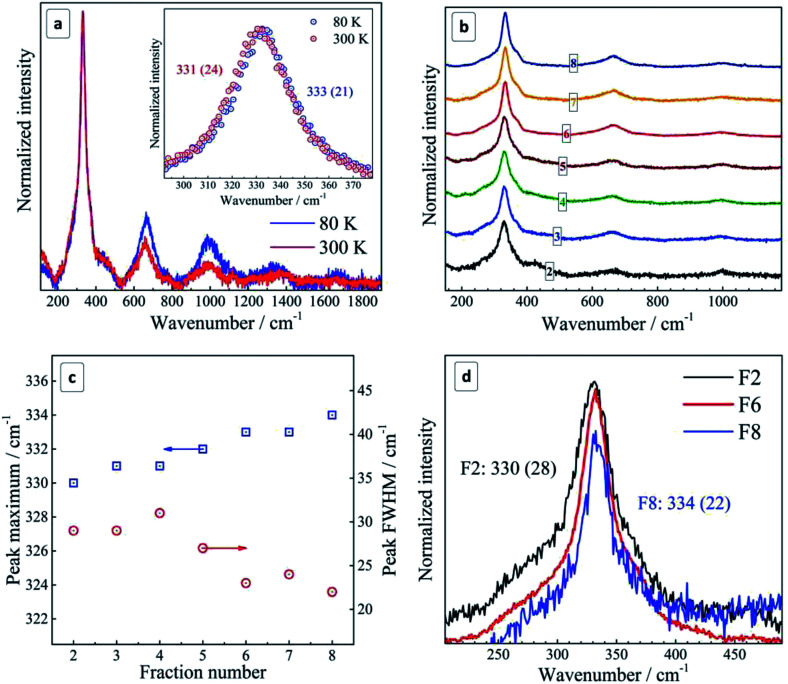
(a) Representative Raman spectra of unfractionated CZTS NCs deposited on Si. Spectra acquired with a resonant excitation *λ*_exc_ = 514.7 nm at 300 K (red curve) and 80 K (blue curve). Inset shows the first-order peak range of the spectra; (b) Raman spectra of size-selected CZTS NCs. (c) Size/fraction-dependence of the position and FWHM of the main Raman peak. (d) Enlarged spectra in the first-order phonon range for fractions F2 (black curve), F6 (red curve), and F8 (blue curve). Spectra were acquired at 300 K during 30 s with 0.1 mW *λ*_exc_ = 514.7 nm. The peak maxima and corresponding FWHM values (in parentheses) are indicated on the figures.

The Raman spectra of the as-prepared unfractionated sample were inspected for NCs deposited on glass, ITO, Si, and Au-coated Si substrates. The representative spectra taken at 300 K and 80 K shown in Fig. 2a were measured on films obtained at optimized deposition conditions that did not lead to the formation of secondary phases and intrinsic defect structures as discussed in the next section. The Raman spectra in Fig. 2a indicate the high crystallinity of the CZTS NCs synthesized at rather mild conditions in water, *i.e.* without using high temperatures and hazardous reagents. The full width at half maximum (FWHM) of the first-order phonon peak at 331–333 cm^−1^ is relatively small, compared to other reports,^13,14,34,39^ only 24 cm^−1^ at room temperature (Fig. 2b). Furthermore, the observed series of multi-phonon peaks recognizable up to the fifth order at ≈1700 cm^−1^ (Fig. 2a) can be taken as another indication of the high quality of the CZTS NC lattice.^50^

By lowering the temperature from 300 K to 80 K, a narrowing and a high frequency shift of the phonon peaks as well as an increased relative intensity of overtones are observed (Fig. 2b), in accordance with expectations for a crystalline semiconductor.^51–53^ The magnitude of the temperature-induced shift, 2 cm^−1^, is smaller than the values of 3–4 cm^−1^ reported for polycrystalline CZTS films.^51^ The latter fact can be related with the notable size distribution in the as-prepared NC ensemble, ≈50% of the average value according to TEM, which contributes to the Raman peak width and may also cause some selectivity of the resonant Raman scattering within the ensemble.^31^ Moreover, the temperature-induced change of the peak width of 3 cm^−1^ in our case is even larger than in ref. 54 and can be partially related to the same effect.

As the Raman measurements performed at low temperature in vacuum did not reveal any anomalous difference to the spectra acquired at room temperature, we obtained an additional proof that the NCs measured at room temperature were not subjected to overheating and structural changes due to laser illumination. At the same time, we have to mention that the frequency position of the main phonon peak in the range reported for bulk CZTS, 337–339 cm^−1^, was observed by us only for very fresh CZTS NC colloids, *i.e.* within about one day after synthesis (ESI, Fig. S4b†). However, because of a strong PL background in these freshly prepared colloids (Fig. S4a†), it was not possible to acquire Raman spectra of a quality sufficient for a detailed analysis. Nevertheless, even in these spectra we could observe an unambiguous trend towards an upward shift of the Raman peak position and its narrowing with increasing fraction number (corresponding to a decrease of the mean NC size) (Fig. S4c†).

The spectra shown in Fig. 2 and analyzed in detail further in this paper were acquired on the thin films prepared from the colloidal solutions by drop-casting. Even though the position of the main Raman peak in these spectra is shifted by about 5 cm^−1^ to lower frequencies compared to the spectra obtained immediately after synthesis, they reveal the same size-dependent trend – an upward frequency shift and a narrowing of this characteristic phonon peak with decreasing NC size. As a possible reason of the frequency shift in the phonon spectra in thin films or even in aged colloids, with respect to freshly prepared colloids, we assume a partial cationic disorder. The factors triggering it and the exact scenario of this structural transformation are beyond the scope of this manuscript. Nevertheless, in the next section we discuss our present experimental evidences of the role of oxidation processes and photostimulated nature of the effect. We would like to stress, however, that the discussed “disorder” is not the one generally applied to solid state notion of the absence of crystallinity. We obviously have to do with a certain rearrangement of the cations (and probably anions) in their sublattice, which does not deteriorate the overall crystalline perfection of the NC, as can be concluded from their sharp phonon spectra (Fig. 2). Most relevant examples of such structural modifications were recently reported by us for stannite CZTS and a so-called “disordered kesterite” modification of CZTS.^6,7^

#### 3.2.2 Phonon spectra of size-selected CZTS NCs

The size-dependent properties of CZTS NCs, particularly the phonon confinement, were studied on the series of samples obtained by fractionating the parental colloidal ensemble as described in the Experimental section. For the Raman measurements, thin films were prepared from fresh solutions by drop casting. The main phonon peak was found to shift to higher frequencies and getting narrower as the CZTS NC size decreased from fraction F2 to F6 and to F8 (Fig. 2b). Both effects are opposite to observations reported for NCs of most other semiconductors which reveal broadening and downward shift of the phonon peaks with decreasing NC size.^31^ However, these effects can be explained by the specifics of the phonon dispersion of the CZTS lattice as described below.

**Fig. 3:**
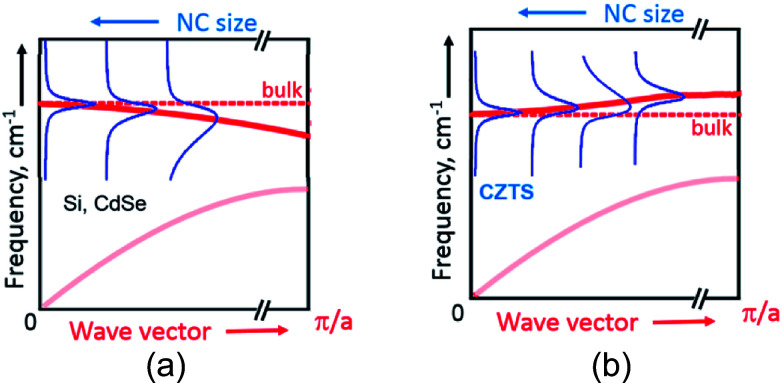
A schematic representation of the phonon confinement-induced downward shift of the Raman peak in case of materials with negative dispersion of optical phonons (a) and the upward shift of in case positive dispersion, as in case of CZTS (b).

Generally, according to the basic phonon confinement model, measuring NCs with a smaller size corresponds to probing larger wavevectors in the dispersion curve of the corresponding bulk crystal. Most of the semiconductors such as Ge, Si, II–VI, III–VI *etc.* possess negative optical phonon dispersions *ω*(*k⃑*), *i.e.* a smaller phonon frequency *ω* for larger wave vectors *k*, and this dispersion becomes more pronounced when moving away from the center of Brillouin zone (*k⃑* = 0).^51^ This property of the bulk dispersion curve is reflected in the Raman spectra of NCs as a downward shift and a broadening of the phonon peak with a decrease of the mean NC size^31^ (Fig. 3a). At the same time, for CZTS^21,55^ and other similar quaternary chalcogenides^25–27^ calculations predict an anomalous phonon dispersion: the frequency increases with *k* in the vicinity of *k⃑* = 0 followed by an almost flat region of *ω*(*k⃑*) (Fig. 3b). Based on this peculiarity of the phonon dispersion we explained earlier the evolution of phonon spectra in bulk Cu_2_ZnSn_*x*_Ge_1−*x*_S_4_ crystals upon varying their composition *x*.^28^

The same anomaly of the phonon dispersion can also account, in our opinion, for the unusual behavior of the phonon Raman peaks with NC size – for smaller NCs the phonon frequency is first increasing from F2 to F6 because of the positive dispersion, while the peak width decreases because the dispersion becomes more flat for larger *k* vectors (corresponding to smaller NC sizes) (Fig. 3b).

Going further to even smaller NCs (from F6 to F8), which apparently correspond to the flat region of the phonon dispersion, produces almost no further peak shift or FWHM change (Fig. 2b and 3b). A broadening of the Raman peaks reported for CZTS NCs with a decreasing mean NC size^13,14^ could be related with the fact that smaller NCs were synthesized at a lower temperature and thus their crystallinity could be poorer, thus resulting in broader phonon peaks. This drawback was avoided in our case as the NCs of all sizes were grown at the same temperature and reactant ratios. Nevertheless, we cannot exclude at the moment contribution to the spectral changes of the different phonon branches and higher-frequency vibrational modes activated by point defects or surface in smaller NCs.^56^ Moreover, the phonon modes with higher wavenumbers can give contribution to the Raman peaks broadening.^57^

### 3.3 Effect of NC film preparation on the formation of Cu_*x*_S phases

#### 3.3.1 Spectral signs of Cu_*x*_S phases

**Fig. 4:**
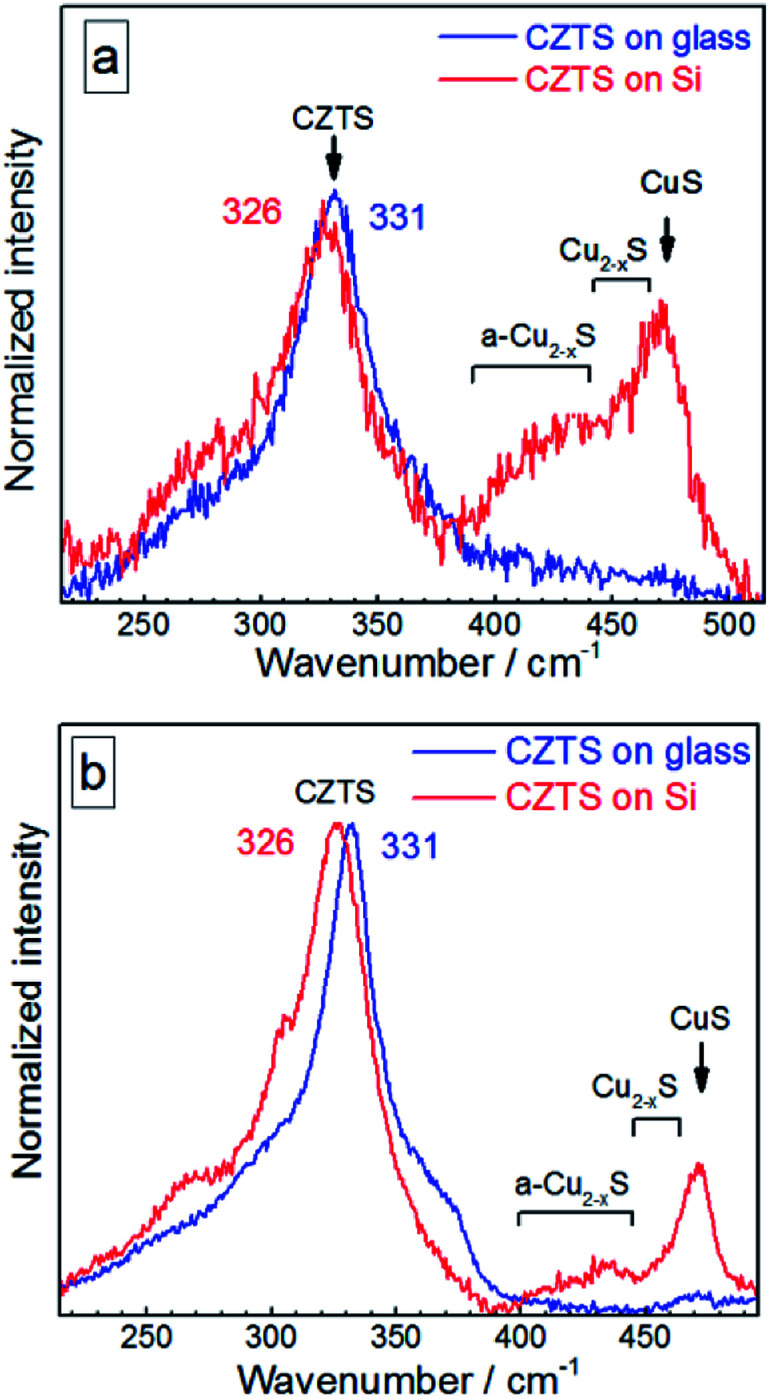
Raman spectra of the initial (not fractionated) CZTS NCs deposited on glass (blue curves) in comparison with the same amount of NC solution deposited on Si substrate (red curves) acquired at 300 K using *λ*_exc_ = 514.7 nm (a) and *λ*_exc_ = 632.8 nm (b).

The Raman spectra of CZTS NCs discussed in the previous section were measured on films formed on a glass substrate by relatively fast (several minutes) drying of the drop-casted solution under ambient air and humidity. At the same time, we noted that the drying of the same amount of NC solution under the same conditions, but on a Si substrate leads to strong additional Raman features between 400 and 500 cm^−1^ and some spectral changes in the range of the CZTS mode at around 250–300 cm^−1^ (Fig. 4).

The distinct maximum at about 472 cm^−1^ can be attributed to the formation of a crystalline Cu_2−*x*_S phase^32,58–61^ observed earlier for CZTS crystals, mainly Cu-rich CZTS.^62–64^ The slight low-frequency asymmetry of this peak can be attributed to variation of *x* in our Cu_2−*x*_S NCs.^32,58–61^ The weaker and broader feature at lower frequencies, peaked at 420–430 cm^−1^, correlates with the Raman spectrum of amorphous Cu_2−*x*_S.^58^ A weak enhancement of the scattering in the range of 250–300 cm^−1^ can also be assigned to the Cu–S bond vibration in the Cu_2−*x*_S phase, because this mode occurs at 265 cm^−1^ with a several times weaker intensity compared to the covalent S–S bond vibration of Cu_2−x_S at 472 cm^−1^.^58–60^

In the case of bulk polycrystalline CZTS films the Cu_2−*x*_S secondary phase was found to be located on the film surface.^24,25^ Therefore, it is reasonable to assume that in the case of our NC samples a Cu_2−*x*_S shell or multiple “islands” can form on the surface of CZTS NCs. The formation of the Cu_2−*x*_S phase (shell or islands) can cause Cu-deficiency in the CZTS NC (core) and thus explain a downward shift and a broadening of the CZTS peak in the spectra with Cu_2−*x*_S features (CZTS on Si, Fig. 4) with respect to the spectra without it (CZTS on glass, Fig. 4). A change of the NC size would cause an opposite shift, as we show above, while a downward shift and broadening of the main CZTS peak as a result of an increased cationic disorder were earlier reported for CZTS (poly)crystals.^7^

The relative intensity of Cu_2−*x*_S-related peaks as compared to the CZTS features is higher if acquired at 514.7 nm excitation (Fig. 4a) than at 632.8 nm excitation (Fig. 4b). This observation can be explained by a resonance of the former excitation with the Cu_2−*x*_S band gap expected at ∼2.5 eV.^64–66^ Another factor determining the difference between the spectra acquired at 514.7 nm and 632.8 nm can be a photostimulated structural transition in CZTS as discussed later in this section.

#### 3.3.2 Influence of the substrate on the Cu_*x*_S-related Raman features

To discriminate a possible effect of the substrate from other factors, such as the drying conditions, we prepared a series of drop-casted NC films on glass, ITO, Si, and Au-coated Si substrates dried in different conditions: (i) left uncovered under ambient conditions, (ii) put under a low vacuum in a desiccator; (iii) kept under the hood; (iv) dried under ambient conditions while covered with a cap of a Petri dish. The details of the sample preparation procedures are provided in the ESI.†

**Fig. 5:**
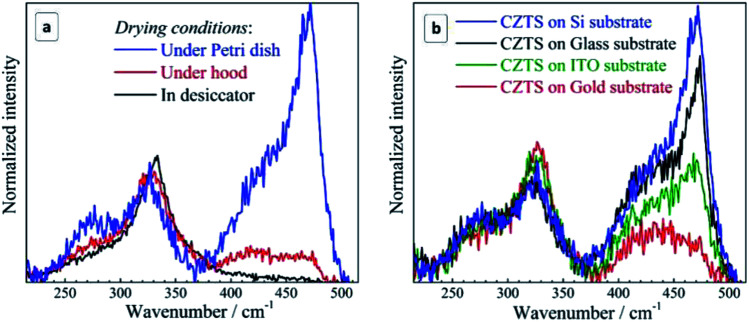
(a) Raman spectra of the non-fractionated CZTS NCs (a) deposited on silicon and dried under different conditions: under/in the hood, under the Petri dish cap, in the desiccator (see text for details) and (b) deposited on different substrates and dried under the Petri dish cap (long drying time, high humidity conditions). Registration conditions: 300 K, *λ*_exc_ = 514.7 nm, exposure of 100 s (a) and 30 s (b).

Compared to case (i), drying is significantly accelerated and no oxygen access is provided in (ii), while in (iii) drying is only slightly accelerated and oxygen access is preserved. In case (iv), drying is significantly slowed down, and occurs at an increased humidity. The results obtained in this experiment for Si substrates are summarized in Fig. 5a. One can see that when reducing the drying time and excluding oxygen in the desiccator no Cu_2−*x*_S peaks are observed at all. Prolonged drying time under the Petri dish cap, on the contrary, enhances the secondary phase peaks dramatically. We found that even on the glass substrate, which caused no Cu_2−*x*_S peaks when drying under normal ambient conditions, the copper–sulfide-related peaks become even stronger than the CZTS one when drying under the Petri dish cap preserving a high humidity of the air confined under the cap.

These observations allow us to conclude that the critical factor for the formation of a secondary Cu_2−*x*_S phase during drying of the CZTS NC solution is the time NCs spend exposed to moisture and oxygen. Furthermore, comparing the lineshape of the spectra in the range of Cu_2−*x*_S vibrations (Fig. 5a), we can conclude that the formation of the secondary phase of copper sulphide starts with amorphous Cu_2−*x*_S (a broad scattering feature peaked at 420–430 cm^−1^) which gradually transforms into crystalline CuS due to increasing number of Cu ions in the 2+ state. Most probably, CZTS NCs exposed to the moisture suffer from a partial hydrolysis resulting in the formation of Sn(OH)_4_, SnO_2_, and intermediate hydrated forms of tin(iv) oxide, while the liberated copper comes into the amorphous Cu_2−*x*_S phase. Such phases are known for their catalytic activity towards oxygen^48^ that can result in the gradual oxidation of Cu(i) into Cu(ii) and the formation of a CuS phase observed in the Raman spectra of the present dried CZTS samples. Copper sulfides were also reported to act as a catalyst of hydrosulfide ion oxidation and, therefore, one can expect them to be active also towards the mercapto-groups of the capping MA ligands. Binding of the thiol ligands to the NC surface is confirmed by core-level XPS spectra of sulphur, showing two pairs of characteristic doublets, corresponding to the NC lattice sulfide (161.1/162.3 eV), and the thiol sulphur bound to the NC surface metal cations (163.2/164.5 eV)^67,68^ (ESI, Fig. S5a†). Additional evidence of the MA bonding to the surface of CZTS NCs was provided by the FTIR spectroscopy that showed characteristic –O–C=O (1585 cm^−1^) and C–H (around 1400 cm^−1^) vibrations of the MA anions (ESI, Fig. S5b†). No peaks at around 2550 cm^−1^ characteristic for S–H vibration^69,70^ can be observed indicating that thiol sulphur of MA is covalently bound to the NC surface. Additionally, the O–C=O band shows a shift to lower energies as compared to individual mercaptoacetic acid (∼1750 cm^−1^),^69,70^ also indicating on the MA binding to the NC surface. Similar spectral effects were earlier reported for MA-stabilized CdS^70^ and CoS^69^ NCs.

The oxidation of surface-bound MA ligands should additionally contribute to the accessibility of the CZTS surface for a deeper hydrolysis and oxidation.

The intensity of copper sulfide-related spectral features depends crucially on the substrate used for the sample preparation. To discriminate the substrate effect we studied four samples produced at the maximum exposure to the ambient air/humidity (dried under Petri dish cap) for the same period. It was found that the intensity of the Cu_2−*x*_S signal is maximal for the silicon substrate and somewhat lower for the glass substrate (Fig. 5b). The sample produced on ITO showed roughly twice lower intensity of the copper–sulfide-related feature. Even lower intensity of this band was observed for the gold substrate.

We assume that the effect of the substrate on the kinetics of the CZTS decomposition also stems from variations in the hydrolysis and oxidation of the surface-anchored CZTS NCs. The NCs are stabilized by bifunctional –OOC–CH_2_–SH anions with the mercapto-group bound to the metal ions on the NC surface and the carboxyl group ionized and contributing to the electrostatic barrier preventing colloidal NCs from the coagulation and precipitation. As the carboxyl groups are prone to form hydrogen bonds with hydroxyl groups on the surface of supporting substrates we may assume that the substrates with a higher hydrophilicity (a higher surface density of OH groups) will facilitate a more even distribution of NCs over the surface, while the substrates with a lower hydrophilicity will favor the aggregation/clustering of NCs on the surface. The latter factor is expected to make the NC surface less accessible to the moisture and air and thus to prevent the NCs from the hydrolysis and oxidation of both capping ligands and Cu(i).

Silicon substrates exposed to the ambient air are typically covered with a silicon oxide layer making such surfaces highly hydrophilic with a density of OH moieties reported to reach 8 groups per nm^2^.^71^ Porous glasses were reported to bear 4–6 OH groups per nm^2^.^72^ Such high concentration of OH groups on the surfaces of glass and silicon favors the even distribution of MA-capped colloidal NCs forming rather smooth films with an average roughness of the order of NC size as we showed earlier for the case of MA-capped silver indium sulfide NCs.^49^

ITO is much less hydrophilic than glass because only a portion of surface In atoms is bound to the OH groups. By studying the adsorption of polyethyleneimine on ITO films the surface density of hydroxyls was estimated as being lower than 1 group per nm^2^.^73^ Because of a much lower surface coverage with OH, the interaction between the carboxyl-terminated MA-capped CZTS NCs and ITO is expected to be much weaker resulting in clustered deposits of agglomerated NCs as exemplified by the SEM image in Fig. S3 (ESI†).

Clean gold surfaces were reported to be hydrophilic, but even a sub-monolayer of adventitious carbonaceous contamination makes it rather hydrophobic.^74^ Only short-lived surface hydroxyl groups were reported to form on the surface of both bulk and nanocrystalline gold and only after a treatment with O_2_ or O_3_.^75,76^

To assess experimentally the hydrophilic/hydrophobic balance on the surface of the studied substrates we measured the contact angle of the drops of both DI water and CZTS ink solution deposited on the same surfaces as those used for the optical measurements. The results are presented in ESI (Fig. S6, Table S1†). We found that the contact angle of both water and NC ink is evidently smaller for the cases of glass and oxidized silicon as compared to the gold and ITO surfaces. The fact indicates a strong difference in the wettability between these two groups of substrates and supporting our above assumptions.

Additionally, we acquired optical images of the CZTS NC inks produced by slow water evaporation on glass and gold in the exact spots where the Raman spectra were taken (ESI, Fig. S7†). It was found that CZTS NC distribute evenly across the surface of glass with no spots or clusterized NCs visible on the millimeter-large areas, while distinct agglomeration of the CZTS NCs and formation of separate spots was observed in the case of gold substrates. These observations support our above assumptions on the effect of the substrate hydrophilicity and the consecutive NC agglomeration on the substrate surface on the formation and intensity of the Cu_2−*x*_S-related spectral features in the Raman spectra of dried CZTS NCs.

#### 3.3.3 Influence of the photoexcitation power on the Cu_*x*_S-related Raman features

**Fig. 6:**
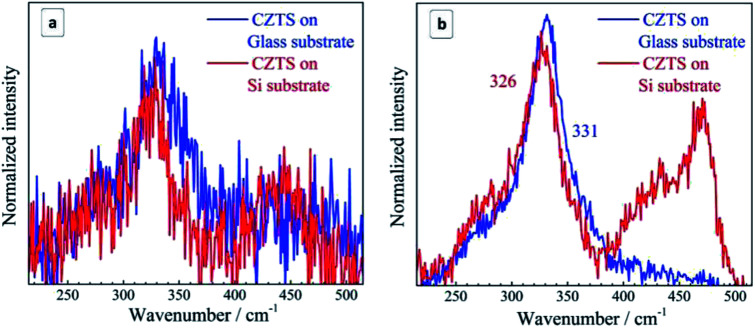
Raman spectra of the CZTS NCs on Si and glass substrates acquired at different power of the 514.7 nm excitation: 0.001 mW (a) and 0.1 mW (b). Exposure is 240 s (a) and 30 s (b).

The suggested mechanism of the partial transformation of CZTS NCs into Cu_2−*x*_S during drying of the samples exposed to a humid environment can also include photoinduced or photostimulated steps/factors. The photoinduced structural transformation recently reported by us for bulk CZTS^6^ can occur under the laser illumination during the Raman measurements. This possibility is illustrated in Fig. 6 by spectra taken at different laser powers of the 514.7 nm excitation which can be strongly absorbed by CZTS. At a laser power of 0.001 mW (Fig. 6a) the weak amorphous copper sulphide feature is detected, while at 0.1 mW also the crystalline CuS peak is present with an intensity comparable to that of the CZTS one (Fig. 6b).

A possible reason for the observed light-induced transformation of CZTS NCs can be the photocatalytic decomposition of the MA ligand shell exposing the NC surface to the hydrolysis. It is well reported that CZTS NCs can be used as photocatalysts for various processes including hydrogen evolution at the expense of the oxidation of sulfide and sulfite ions.^77,78^

In the present case similar processes can take place, that is, the MA oxidation resulting from the transfer of valence band holes to the MA ligands with photogenerated conduction band electrons captured by air oxygen. The photogenerated holes can also oxidize lattice sulfide ions, similar as it happens during the oxidative photocorrosion of metal chalcogenide NCs. This process is expected to expose Sn(iv) sites to the attack by water resulting in the hydrolytic decomposition of the CZTS lattice and the isolation of copper in the form of a separate Cu_2−*x*_S phase. Additionally, the photogenerated holes can contribute to the oxidation of lattice Cu(i) into Cu(ii) favoring the formation of a CuS phase clearly observed at intense photoexcitation (Fig. 6b).

## 4 Conclusions

We reported a Raman study of Cu_2_ZnSnS_4_ NCs produced by a “green” synthesis in aqueous solutions and stabilized by mercaptoacetate anions. A series of size-selected CZTS NCs was isolated by a controlled precipitation from such colloidal ensemble for a size-dependent Raman study. As all these CZTS NCs of different mean size were produced in the same conditions (temperature, capping ligand, *etc.*) we can discriminate the size effect from those related to the NC composition, phase, and surface chemistry.

The size-selected CZTS NCs thus obtained showed a phonon confinement effect, with the frequency position of the main phonon shifted by about 4 cm^−1^ between 3 nm and 2 nm NC diameters, accompanied by a narrowing of the main phonon feature. The upward shift of the main phonon frequency with the NC size decrease is assumed to result from an anomalous positive phonon dispersion curves of kesterites. Narrowing of the Raman peak can originate from the peculiar shape of the phonon dispersion in CZTS, and partially by a reduction of the NC size dispersion upon each fractioning step.

The conditions of the preparation of samples for optical studies, the nature of the support of the dried CZTS NC film, as well as the conditions of the registration of Raman spectra were found to affect crucially the spectral properties of the studied samples highlighting these factors as possible origins for the scatter of reported results on the vibrational properties of kesterite NCs published by various groups. In particular, we found that the drying conditions of the kesterite NC films can strongly affect the phase composition of the samples resulting in a partial oxidation and hydrolysis of CZTS NCs and the formation of additional Cu_2−*x*_S and CuS phases with characteristic Raman signatures. The composition and relative amount of such admixture phases depend on the drying rate, the humidity of environment as well as on the presence/absence of air oxygen. The downward shift and broadening of the CZTS peaks correlating with the intensity of the Cu_2−*x*_S/CuS peaks can be explained by increased cationic disorder in the host (CZTS) lattice at a higher content of the secondary phase.

The support nature was found to strongly affect the amount of admixture copper sulfide phases in the same sample preparation conditions, the Cu_2−*x*_S/CuS content being the highest for oxidized silicon and glass and declining strongly for ITO and gold supports. The effect was assumed to originate from a different hydrophilicity of the support surface, more hydrophilic Si/SiO_2_ and glass surfaces favoring a more even distribution of CZTS NCs and their better accessibility to the oxidation/hydrolysis in the ambient humid air.

The excitation light intensity was also found to affect the samples, the copper sulfide content increasing with an increase of the laser power, most probably, due to the possibility of the photocatalytic oxidation of surface ligands and lattice copper and sulfide by the charge carriers photogenerated in the CZTS NCs.

## Conflicts of interest

There are no conflicts to declare.

## Supplementary Material

RA-008-C8RA05390A-s001

## References

[cit1] Green M. A., Hishikawa Y., Warta W., Dunlop E. D., Levi D. H., Hohl-Ebinger J., Ho-Baillie A. W. H. (2017). Prog. Photovoltaics.

[cit2] Wallace S. K., Mitzi D. B., Walsh A. (2017). ACS Energy Lett..

[cit3] Sandroni M., Wegner K. D., Aldakov D., Reiss P. (2017). ACS Energy Lett..

[cit4] Kumar M., Dubey A., Adhikari N., Venkatesan S., Qiao Q. (2015). Energy Environ. Sci..

[cit5] Baranowski L. L., Zawadzki P., Lany S., Toberer E. S., Zakutayev A. (2016). Semicond. Sci. Technol..

[cit6] Valakh M. Y., Dzhagan V. M., Babichuk I. S., Fontane X., Perez-Rodriquez A., Schorr S. (2013). JETP Lett..

[cit7] Valakh M. Y., Kolomys O. F., Ponomaryov S. S., Yukhymchuk V. O., Babichuk I. S., Izquierdo-Roca V., Saucedo E., Perez-Rodriguez A., Morante J. R., Schorr S., Bodnar I. V. (2013). Phys. Status Solidi RRL.

[cit8] Tan J. M., Lee Y. H., Pedireddy S., Baikie T., Ling X. Y., Wong L. H. (2014). J. Am. Chem. Soc..

[cit9] Dimitrievska M., Boero F., Litvinchuk A. P., Delsante S., Borzone G., Perez-Rodriguez A., Izquierdo-Roca V. (2017). Inorg. Chem..

[cit10] V Kovalenko M., Manna L., Cabot A., Hens Z., V Talapin D., Kagan C. R., Klimov X. V. I., Rogach A. L., Reiss P., Milliron D. J., Guyot-Sionnnest P., Konstantatos G., Parak W. J., Hyeon T., Korgel B. A., Murray C. B., Heiss W. (2015). ACS Nano.

[cit11] Pietryga J. M., Park Y. S., Lim J., Fidler A. F., Bae W. K., Brovelli S., Klimov V. I. (2016). Chem. Rev..

[cit12] Freitas J. N., Gonçalves A. S., Nogueira A. F. (2014). Nanoscale.

[cit13] Khare A., Wills A. W., Ammerman L. M., Norris D. J., Aydil E. S. (2011). Chem. Commun..

[cit14] Liu W. C., Guo B. L., Wu X. S., Zhang F. M., Mak C. L., Wong K. H. (2013). J. Mater. Chem. A.

[cit15] Zhou M., Gong Y., Xu J., Fang G., Xu Q., Dong J. (2013). J. Alloys Compd..

[cit16] Jing L., Kershaw S. V., Li Y., Huang X., Li Y., Rogach A. L., Gao M. (2016). Chem. Rev..

[cit17] Raevskaya A., Rosovik O., Kozytskiy A., Stroyuk O., Dzhagan V., Zahn D. R. T. (2016). RSC Adv..

[cit18] Raevskaya A., Lesnyak V., Haubold D., Dzhagan V., Stroyuk O., Gaponik N., Zahn D. R. T., Eychmüller A. (2017). J. Phys. Chem. C.

[cit19] Yokoyama D., Minegishi T., Jimbo K., Hisatomi T., Ma G., Katayama M., Kubota J., Katagiri H., Domen K. (2010). Appl. Phys. Express.

[cit20] Wang J., Zhang P., Song X., Gao L. (2014). RSC Adv..

[cit21] Khare A., Himmetoglu B., Johnson M., Norris D. J., Cococcioni M., Aydil E. S. (2012). J. Appl. Phys..

[cit22] Grosvenor A. P., Biesinger M. C., Smart R. S. C., McIntyre N. S. (2006). Surf. Sci..

[cit23] Fernandes P. A., Salomé P. M. P., Da Cunha A. F. (2011). J. Alloys Compd..

[cit24] Dimitrievska M., Fairbrother A., Pérez-Rodríguez A., Saucedo E., Izquierdo-Roca V. (2014). Acta Mater..

[cit25] Valakh M. Y., Litvinchuk A. P., Dzhagan V. M., Yukhymchuk V. O., Yaremko A. M., Romanyuk Y. A., Guc M., V Bodnar I., Pérez-Rodríguez A., Zahn D. R. T. (2016). J. Phys.: Condens. Matter.

[cit26] Litvinchuk A. P., Dzhagan V. M., Yukhymchuk V. O., Valakh M. Y., Parasyuk O. V., Piskach L. V., Wang X., Jacobson A. J., Zahn D. R. T. (2016). Phys. Status Solidi B.

[cit27] Litvinchuk A. P., Dzhagan V. M., Yukhymchuk V. O., Valakh M. Y., Babichuk I. S., Parasyuk O. V., Piskach L. V., Gordan O. D., Zahn D. R. T. (2014). Phys. Rev. B.

[cit28] Valakh M. Y., Litvinchuk A. P., Dzhagan V. M., Yukhymchuk V. O., Havryliuk Y. O., Guc M., Bodnar I. V., Izquierdo-Roca V., Pérez-Rodríguez A., Zahn D. R. T. (2016). RSC Adv..

[cit29] Dimitrievska M., Fairbrother A., Fontané X., Jawhari T., Izquierdo-Roca V., Saucedo E., Pérez-Rodríguez A., Dimitrievska M., Fairbrother A., Saucedo E. (2014). Appl. Phys. Lett..

[cit30] Chesman A. S. R., van Embden J., Duffy N. W., Webster N. a S., Jasieniak J. J., Van Embden J. (2013). Cryst. Growth Des..

[cit31] Dzhagan V. M., Valakh M. Y., Raevskaya A. E., Stroyuk A. L., Kuchmiy S. Y., Zahn D. R. T. (2008). Nanotechnology.

[cit32] Yeryukov N. A., Milekhin A. G., Sveshnikova L. L., Duda T. A., Pokrovsky L. D., Gutakovskii A. K., Batsanov S. A., Rodyakina E. E., Latyshev A. V., Zahn D. R. T. (2014). J. Phys. Chem. C.

[cit33] Dzhagan V. M., Litvinchuk A. P., Kruszynska M., Kolny-Olesiak J., Valakh M. Y., Zahn D. R. T. (2014). J. Phys. Chem. C.

[cit34] Flynn B., Wang W., Chang C. H., Herman G. S. (2012). Phys. Status Solidi.

[cit35] Mainz R., Singh A., Levcenko S., Klaus M., Genzel C., Ryan K. M., Unold T. (2014). Nat. Commun..

[cit36] Singh A., Singh S., Levcenko S., Unold T., Laffir F., Ryan K. M. (2013). Angew. Chem., Int. Ed. Engl..

[cit37] Tosun B. S., Chernomordik B. D., Gunawan A. A., Williams B., Mkhoyan K. A., Francis L. F., Aydil E. S. (2013). Chem. Commun..

[cit38] Hlaing Oo W. M., Johnson J. L., Bhatia A., Lund E. A., Nowell M. M., Scarpulla M. A. (2011). J. Electron. Mater..

[cit39] Wang X., Sun Z., Shao C., Boye D. M., Zhao J. (2011). Nanotechnology.

[cit40] Sheng H., Wenjun L., Zhigang Z. (2013). J. Phys. D: Appl. Phys..

[cit41] Babichuk I. S., Yukhymchuk V. O., Dzhagan V. M., Valakh M. Y., Leon M., Yanchuk I. B., Gule E. G., Greshchuk O. M. (2013). Funct. Mater..

[cit42] Ge J., Yu W., Cao H., Jiang J., Ma J., Yang L., Yang P., Hu Z., Chu J. (2012). Phys. Status Solidi.

[cit43] Zou C., Zhang L., Lin D., Yang Y., Li Q., Xu X., Chen X., Huang S. (2011). CrystEngComm.

[cit44] Chem J. M., Jiang H., Dai P., Feng Z., Fan W., Zhan J. (2012). J. Mater. Chem..

[cit45] Flynn B., Wang W., Chang C. H., Herman G. S. (2012). Phys. Status Solidi.

[cit46] Park J., Song M., Jung W. M., Lee W. Y., Kim H., Kim Y., Hwang C., Shim I.-W. (2013). Dalton Trans..

[cit47] Singh A., Geaney H., Laffir F., Ryan K. M. (2012). J. Am. Chem. Soc..

[cit48] Raevskaya A. E., Stroyuk A. L., Kuchmii S. Y., Kryukov A. I. (2004). . Mol. Catal. A: Chem..

[cit49] Stroyuk O., Raevskaya A., Spranger F., Selyshchev O., Dzhagan V., Schulze S., Zahn D. R. T., Eychmüller A. (2018). J. Phys. Chem. C.

[cit50] Valakh M. Y., Litvinchuk A. P. (1985). Soviet Physics-Solid State.

[cit51] Loudon R. (1964). Adv. Phys..

[cit52] Dzhagan V., Valakh M. Y., Kolny-Olesiak J., Lokteva I., Zahn D. R. T. (2009). Appl. Phys. Lett..

[cit53] Dzhagan V. M., Valakh M. Y., Himcinschi C., Milekhin A. G., Solonenko D., Yeryukov N. A., Raevskaya O. E., Stroyuk O. L., Zahn D. R. T. (2014). J. Phys. Chem. C.

[cit54] Diamond A. M., Corbellini L., Balasubramaniam K. R., Chen S., Wang S., Matthews T. S., Wang L. W., Ramesh R., Ager J. W. (2012). Phys. Status Solidi A.

[cit55] Jackson A. J., Walsh A. (2014). J. Mater. Chem. A.

[cit56] Kosyak V., Mortazavi Amiri N. B., Postnikov A. V., Scarpulla M. A. (2013). J. Appl. Phys..

[cit57] Rolo A. G., Vasilevskiy M. I. (2007). J. Raman Spectrosc..

[cit58] Munce C. G., Parker G. K., Holt S. A., Hope G. A. (2007). Colloids Surf., A.

[cit59] Kumar P., Nagarajan R. (2011). Inorg. Chem..

[cit60] Cruz J. S., Hernández S. A. M., Hernández J. J. C., Rodríguez R. M., Pérez R. C., Delgado G. T., Sandoval S. J. (2012). Chalcogenide Lett..

[cit61] Ishii M., Shibata K., Nozaki H. (1993). J. Solid State Chem..

[cit62] Yoo H., Kim J. (2010). Thin Solid Films.

[cit63] Xu J., Yang X., Yang Q. D., Wong T. L., Lee C. S. (2012). J. Phys. Chem. C.

[cit64] Silvester E. J., Grieser F., Sexton B. A., Healy T. W. (1991). Langmuir.

[cit65] Grozdanov I., Najdoski M. (1995). J. Solid State Chem..

[cit66] Kumar P., Nagarajan R., Sarangi R. (2013). J. Mater. Chem. C.

[cit67] Raevskaya A. E., Stroyuk O. L., Panasiuk Y. V., Dzhagan V. M., Solonenko D. I., Schulze S., Zahn D. R. T. (2018). Nano-Struct. Nano-Objects.

[cit68] Castner D. G., Hinds K., Grainger D. W. (1996). Langmuir.

[cit69] Congiu M., Lanuti A., di Carlo A., Graeff C. F. O. (2015). Sol. Energy.

[cit70] Vikraman A. E., Jose A. R., Jacob M., Kumar K. G. (2015). Anal. Methods.

[cit71] McCafferty E., Wightman J. P. (1998). Surf. Interface Anal..

[cit72] Zhuravlev L. T. (1987). Langmuir.

[cit73] Ok C., Hong S., Kim M., Park S., Won J. (2004). J. Colloid Interface Sci..

[cit74] Smith T. (1980). J. Colloid Interface Sci..

[cit75] Gong J., Mullins C. B. (2009). Acc. Chem. Res..

[cit76] Quiller R. G., Baker T. A., Deng X., Colling M. E., Min B. K., Friend C. M. (2008). J. Chem. Phys..

[cit77] Zhang K., Guo L. (2013). Catal. Sci. Technol..

[cit78] Regulacio M. D., Han M.-Y. (2016). Acc. Chem. Res..

