# Supplementary material for: Raman characterization of Cu2ZnSnS4 nanocrystals: phonon confinement effect and formation of CuxS phases
Source: RSC Adv. 2018 Aug 31;8(54):30736–46. doi: 10.1039/c8ra05390a (PMC9085493; doi:10.1039/c8ra05390a)
Supplement: RA-008-C8RA05390A-s001 [file RA-008-C8RA05390A-s001.pdf]

Electronic Supplementary Material (ESI) for RSC Advances.
This journal is © The Royal Society of Chemistry 2018

Electronic Supporting Information

Raman characterization of Cu_2ZnSnS_4 nanocrystals: phonon confinement effect and the role of the deposition conditions on $Cu_{2-x}S$-phase formation

Ye. Havryliuk[1], M.Ya. Valakh[1], V. Dzhagan[1], O. Greshchuk[1], V. Yukhymchuk[1], A. Raevskaya[2,3], O. Stroyuk[2,3], O. Selyshchev[4], N. Gaponik[3], D.R.T. Zahn[4]

[1]*V. E. Lashkaryov Institute of Semiconductors Physics, Nat. Acad. of Sci. of Ukraine, 03028 Kyiv, Ukraine*

[2]*L.V . Pysarzhevsky Institute of Physical Chemistry, Nat. Acad. of Sci. of Ukraine, 03028 Kyiv, Ukraine*

[3]*Physical Chemistry, TU Dresden, 01062 Dresden, Germany*

[4]*Semiconductor Physics, Chemnitz University of Technology, 09107 Chemnitz, Germany*

Description of the preparation of samples for the studies of influences of substrate and drying conditions on the Raman spectra of CZTS NCs. To discriminate a possible effect of the substrate from other factors, such as the drying conditions, we prepared a series of drop-casted NC films on glass, ITO, Si, and Au-coated Si substrates dried in different conditions: (i) under ambient conditions uncovered, (ii) under low-vacuum in a desiccator; (iii) under the air circulating hood; (iv) under ambient conditions, covered with a cap of Petri dish.

Substrates.

Glass slides (float glass) were manufactured by VWR, cleaned consecutively with ethanol and water under ultrasonic treatment in laboratory ultrasonic bath, and cut into 1×1 cm^2 pieces for the sample preparations. 0.1 mL CZTS solutions were drop-cased onto glass substrates and left for drying in the dark in different conditions.

ITO (indium tin oxide) was supplied by Sigma Aldrich and cleaned consecutively with ethanol and water in aqueous ultrasonic bath. The ITO plates were cut into 1×1 cm^2 pieces on which 0.1 mL of CZTS solutions were drop-casted and left for drying.

Silicon wafers (Si(111), p-type) were produced by Silchem GbmH (Friberg, Germany), cleaned with ethanol and with water in ultrasonic bath. 0.1 mL of CZTS solutions were drop-cased onto 1×1 cm^2 silicon pieces and left for drying in the dark. The silicon wafers comprised a natural surface oxidized layer of around 10-nm of SiO^2 and therefore the actual drying of CZTS colloids occurred on the hydrophilic silica surface.

Si/Au substrates were produced by the magnetron sputtering of the metallic Au on Si wafers, the Au layer was around 100 nm thick. The Si/Au substrates with an area of 1×1 cm^2 were kept under ambient conditions and have not been subjected to additional cleaning procedures. 0.1 mL of CZTS solutions were drop-casted onto Si/Au substrates with an area of 1×1 cm^2 and left for drying in the dark.

Drying procedures. Identical samples were used in different drying procedures, produced by maintaining equal conditions for all substrates and colloids probed.

A set of samples (i) was produced on glass, ITO, Si/SiO_2 and Si/Au at the ambient air circulation. The samples were left for natural drying for 24 h.

To increase the drying speed and to minimize the effects of humidity and oxygen from the air on properties of CZTS a set of samples (ii) were drying in a desiccator under vacuum (pressure ~10 mmHg) and with concentrated sulfuric acid (98%) as moisture absorbing agent. The pumping was carried out by a LPG motor pump (Elnor Motors) until the complete drying occurred (~2 hours). Then sulfuric acid was replaced with silica gel, and the samples were kept under static vacuum until the measurements starting.

To assess the role of air and lowered humidity during the drying of the CZTS a set of samples (iii) was dried under a laboratory hood with circulating air. The hood was 1 m^3 in volume and pumped with the air with an approximate rate of 10 L per h. The drying was performed for 24 h before the optical measurements.

To retard the drying as much as possible and assess the role of humidity on the optical properties of CZTS NCs a set of samples (iv) were dried at elevated moisture. The latter was achieved by covering the drying samples with a cap of Petri dish. The set of samples with an area of each 1×1 cm^2 were covered with the glass Petri dish caps with a diameter of 10 cm and height of 1.5 cm. To release moisture, the edge of the Petri dish was raised by ~ 2 mm (thickness of two glass slides). The samples were left for 96 h till the complete drying occurred in this confined volume.

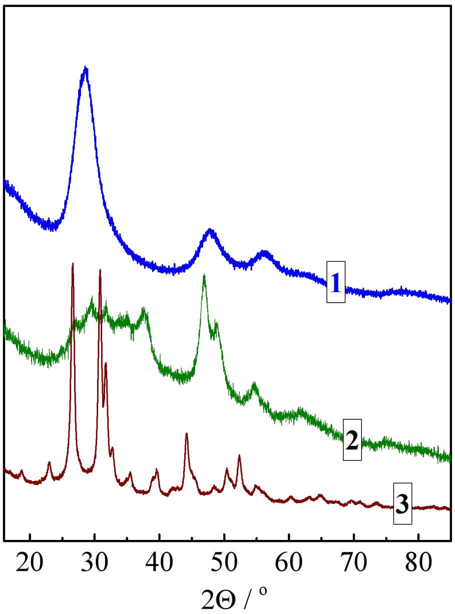

Fig. S1. X-ray diffraction patterns of Cu-Zn-Sn-S (curve 1), Cu-S (2), and Sn–S (3) phases produced via the interaction of the corresponding MA complexes with Na_2S.

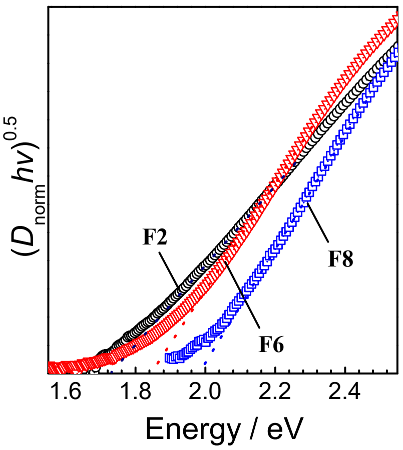

Fig. S2. Absorption spectra of size-selected CZTS NCs from fractions F2, F6 and F8 presented in Tauc coordinates.

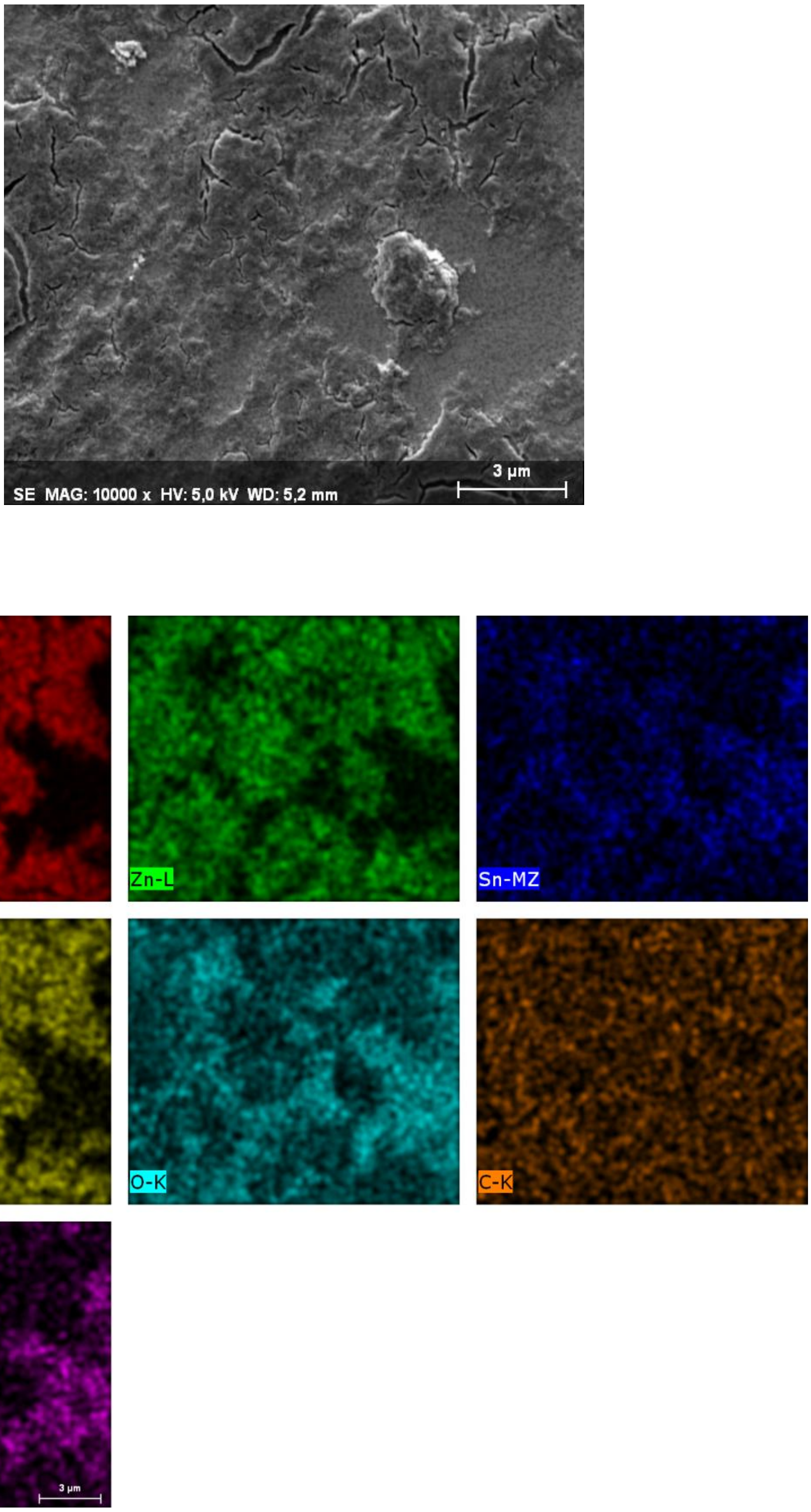

Fig. S3. SEM image (top) and mapping of Cu, Zn, Sn, S, O, C, and In across the samples by EDX measurements.

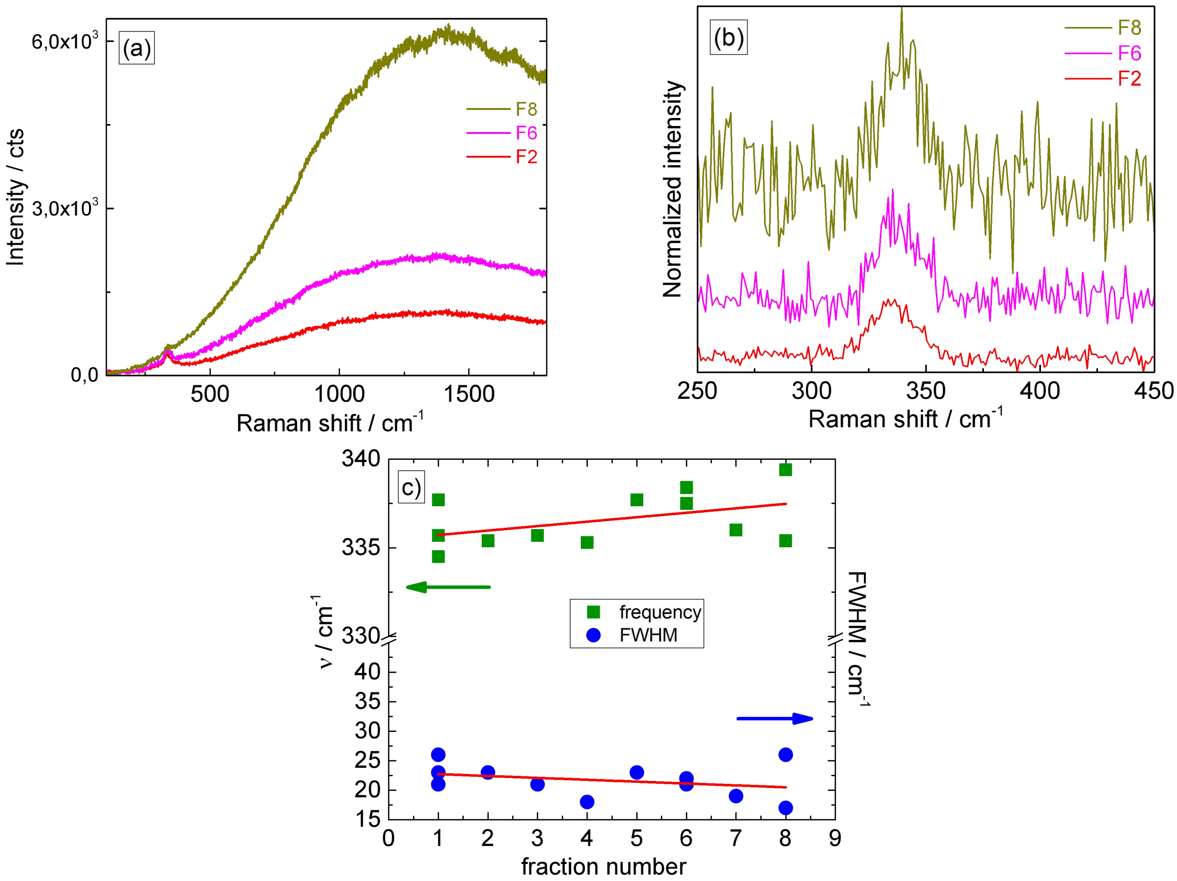

Fig. S4. (a) Representative raw Raman spectra of the freshly prepared size-separated series of NCs in solution; (b) the same spectra after subtraction of the PL background; (c) peak position (green squares) and widths (blue circles) obtained from a single peak fitting, along with the corresponding linear fits (red lines). λ_{exc} = 514.7 nm, 0.1 mW, T=300 K.

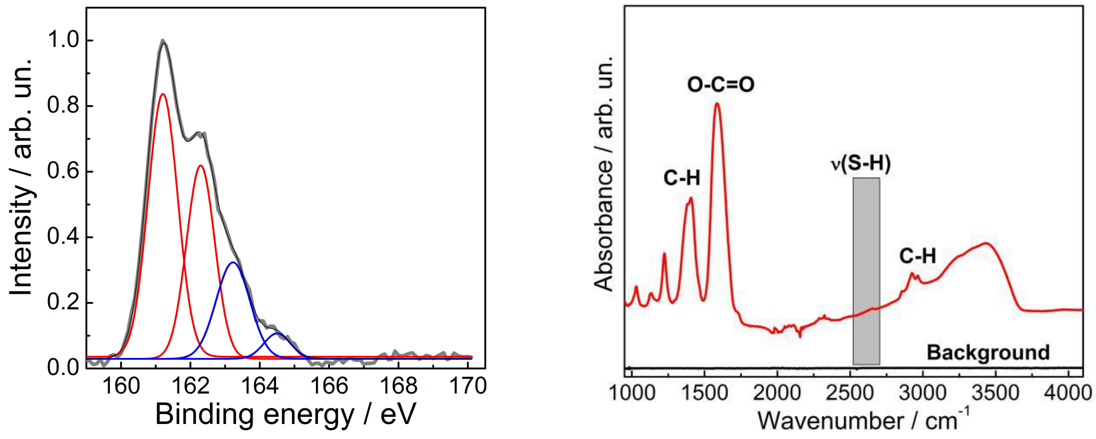

a b

Fig. S5. (a) High-resolution XPS spectrum of CZTS NCs in the range of S 2p electron binding energies. (b) FTIR absorption spectrum of CZTS NCs capped with MA.

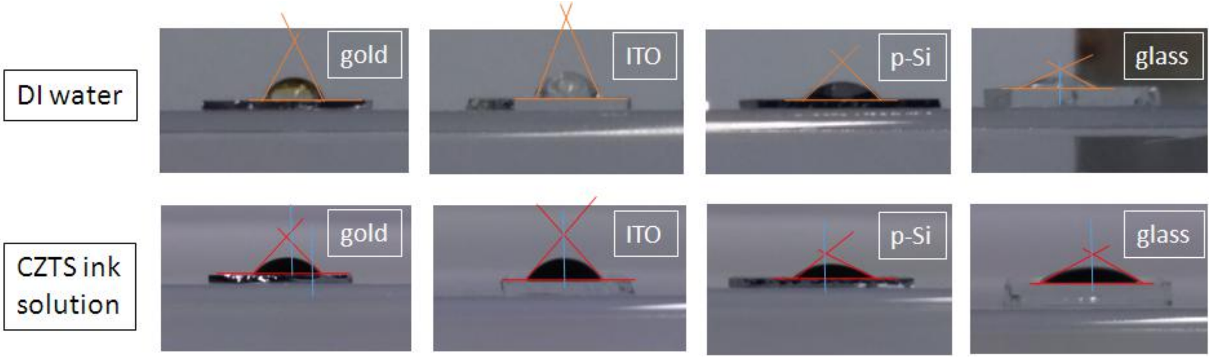

Fig. S6. Photographs of drops of DI water (upper panel) and CZTS NC ink (lower panel) placed on the surface of gold, ITO, oxidized silicon and glass (from left to right images, respectively). Read lines serve as eye guides for the determination of the contact angles.

Table S1. Contact angle for DI water and CZTS NC ink drops on various substrates determined from the photographs presented in Fig. S3. The angles were measured twice from two different surface spots and drops (α_1 and α_2) and averaged producing $\bar{\alpha}$.

Substrate	DI water			CZTS ink water solution		
	α_1, °	α_2, °	$\bar{\alpha}$, °	α_1, °	α_2, °	$\bar{\alpha}$, °
Gold	62 ± 2	64 ± 2	63 ± 2	49 ± 2	47 ± 2	48 ± 2
ITO	68 ± 2	68 ± 2	68 ± 2	49 ± 2	49 ± 2	49 ± 2
p-Si	46 ± 2	48 ± 2	47 ± 2	37 ± 4	28 ± 4	33 ± 4
glass	23 ± 4	25 ± 4	24 ± 4	28 ± 4	28 ± 4	28 ± 4

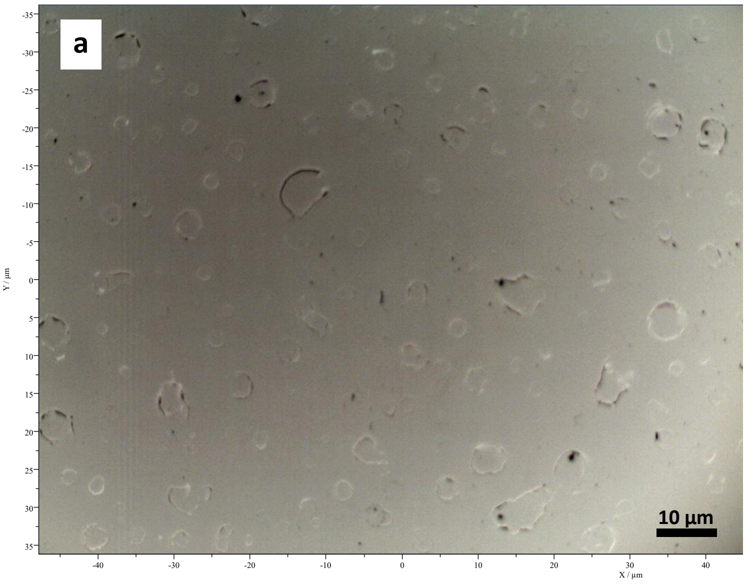

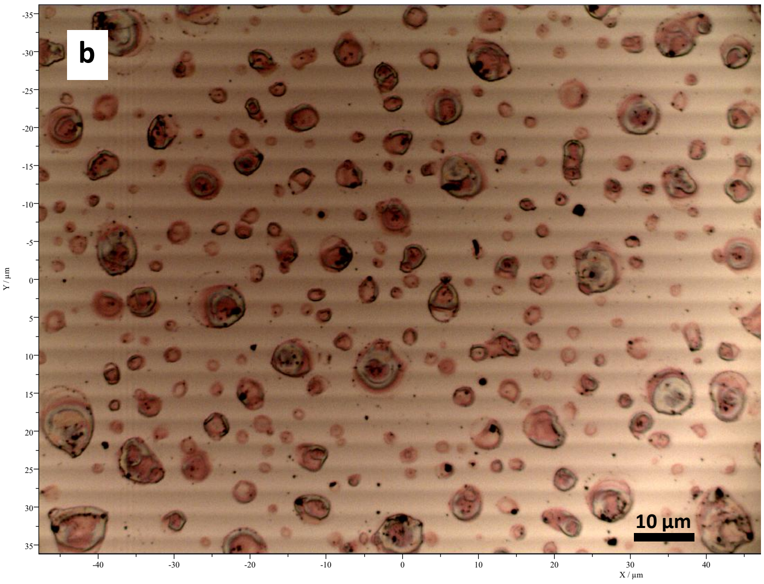

Fig. S7. Images of dried CZTS NC ink spots on glass (a) and gold (b) taken by the LabRam HR800 confocal microscope immediately before the registration of Raman spectra.
